# Deciphering centuries of selection: genetic structure and reproductive screening in Basilicata and Calabria’s ancient olives

**DOI:** 10.3389/fpls.2026.1887538

**Published:** 2026-08-12

**Authors:** Roberto Mariotti, Filomena Carriero, Thomas Vatrano, Giovanni Lacertosa, Roya Nikbakht, Muhammad Ajmal Bashir, Lorenzo Cruciani, Soraya Mousavi

**Affiliations:** 1 National Research Council, Institute of Biosciences and Bioresources, Perugia, Italy; 2 Agenzia Lucana di Sviluppo e di Innovazione in Agricoltura (ALSIA) - Metapontum Agrobios Research Center, Metaponto, Italy; 3 Freelancer Agronomist, Borgia, Italy

**Keywords:** *GA2ox*-S, genetic diversity, monumental trees, *Olea europaea* L., self-incompatibility

## Abstract

The olive tree (*Olea europaea* L.) is a cornerstone of Mediterranean biocultural heritage, yet the shift toward intensive monoculture threatens its vast genetic diversity. This study presents a comprehensive molecular characterization of 347 olive accessions, including 172 monumental trees, from the Calabria and Basilicata regions of Southern Italy. Utilizing 10 nuclear SSR markers and chloroplast (cp-SSR) genotyping, we explored genetic diversity and maternal inheritance. Furthermore, we employed a novel diagnostic marker targeting the *GA2ox*-S gene, the functional determinant of the diallelic self-incompatibility (DSI) system, to assign genotypes to reproductive groups G1 and G2. Our results identified 114 unique genotypes, representing a significant reservoir of uncatalogued traditional germplasm. Notably, we discovered three instances of putative polyploidy in indigenous Calabrian olives, a rarity for the species typically considered diploid. Comparative analysis with an international dataset revealed a highly admixed genetic structure, with local genotypes showing strong affinity to Algerian and Greek pools while remaining distinct from Western Mediterranean and Middle Eastern clusters. Systematic genotyping of both canopies and rootstocks provided genetic evidence consistent with historical grafting practices in 34 instances. We observed a clear structural and maternal partition: cultivated canopies predominantly belonged to the E1 (Eastern) lineage, while uncultivated rootstocks preserved the indigenous E2 (Central-Western) maternal heritage. Reproductive screening revealed a regional dichotomy: the G1 group dominates in Basilicata, while the G2 group is more prevalent among Calabrian landraces. These findings underscore how historical agricultural practices, grafting elite clones onto resilient wild oleasters, clonal propagation, and DSI-driven obligate outcrossing have synergistically shaped the high heterozygosity of Southern Italian groves. This work provides valuable genetic resources for conservation and future breeding programs aimed at exploring resilience against climate change and emerging pathogens.

## 1 Introduction

The olive tree (*Olea europaea* L.) is an iconic and economically vital crop of the Mediterranean Basin, characterized by a rich genetic heritage comprising thousands of cultivars and local ecotypes (Bazakos et al., 2023; Mariotti et al., 2023). Monumental olive trees serve as important genetic reservoirs harboring unique genetic and epigenetic traits related to stress tolerance, longevity, and local adaptation (Barazani et al., 2014; Petruccelli et al., 2021; Palli et al., 2023; Valeri et al., 2022). The modern trend of restricting cultivation to a highly limited number of varieties threatens this diversity and exacerbates the risk of genetic erosion, especially in the face of future climate change scenarios (Mariotti et al., 2016). Historical and physical evidence underscores the deep roots of olive cultivation in southern Italy; for instance, an olive tree in Ferrandina (Basilicata), has been scientifically radiocarbon-dated to 680 ± 57 years old, standing as testaments to centuries of continuous agricultural management (Palli et al., 2023). Grafting, a practice established since antiquity, was a key driver in olive cultivation, allows to propagate elite cultivars, often with larger fruits or higher oil content, onto resilient wild oleasters or locally adapted rootstocks (Barazani et al., 2014; Sales et al., 2021; Valeri et al., 2022). Nuclear simple sequence repeat (SSR) markers and chloroplast polymorphisms can be used to investigate historical grafting practices (Mousavi et al., 2024; Mariotti et al., 2023). Notably, the analysis of distinct maternal lineages has revealed that while the E1 chlorotype is predominant globally due to an eastern Mediterranean origin and spread, the E2 and E3 lineages are typically associated with central-western Mediterranean autochthonous germplasm. Analyzing canopies and rootstocks separately reveals genetic evidence consistent with historical grafting between these distinct lineages, shedding light on ancient selection practices and human-mediated gene flow (Mariotti et al., 2023; Mousavi et al., 2024). Beyond vegetative propagation, the genetic structure of olive populations is profoundly shaped a homomorphic diallelic self-incompatibility (DSI) system that influences sexual reproduction (Castric et al., 2024; Mariotti et al., 2021). This biological mechanism restricts successful fertilization to crosses between individuals belonging to two distinct compatibility groups, typically designated as G1 and G2 (or [Ha] and [Hb]) (Castric et al., 2024; Mariotti et al., 2020). Molecular markers now enable reliable identification and prediction of these reproductive groups (Mariotti et al., 2020).

Italy hosts a significant portion of the world’s known olive germplasm, with southern regions like Calabria and Basilicata exhibiting highly structured and variegated genetic pools (Muzzalupo et al., 2014; Marra et al., 2013). Archaeological evidence from the Late Bronze Age confirms that the exploitation of wild oleasters and local olive oil production were already established in Calabria centuries before the arrival of Greek colonists (Attema, 2008; Lentjes, 2013). Systematic cultivation expanded rapidly with the 8th-century BC Greek colonies (as Sibari and Crotone), reaching such high agricultural prestige that Roman authors like Pliny the Elder attributed the “cultural paternity” of the olive in Europe to the Calabrian region (Attema, 2008; Lentjes, 2013). Unlike more isolated populations, Calabrian and neighboring mainland populations exhibit a highly intermixed genetic structure, reflecting frequent and dynamic historical exchanges (Muzzalupo et al., 2014; Marra et al., 2013). Historical documents and genetic analyses confirm that this agricultural landscape was actively shaped by the continuous transfer of plant material; for example, the variety ‘Ogliarola del Vulture’, characteristic of northeastern Basilicata, was imported from Calabria during the 18th century (Carriero et al., 2002). Moreover, historical traditions, such as the establishment of Benedictine monasteries in Montescaglioso (Basilicata) in the 1300s, may have influenced germplasm dispersal, as recent genetic evidence surprisingly suggests that major central Italian varieties like ‘Frantoio’ may be clones of southern varieties like ‘Ogliarola del Bradano’ (Carriero et al., 2002). Beyond human-mediated movement, local diversity in these regions has also been driven by sexual reproduction and somatic mutations; SSR-based parentage simulations have identified direct parent-sibling relationships between local Calabrian varieties (e.g., ‘Carolea’ and ‘Tombarella’), as well as point mutations generating intra-varietal diversity within regional groups like ‘Ottobratica’ (Marra et al., 2013).

Despite existing surveys of Italian germplasm, a targeted, fine-scale exploration of the genetic diversity, maternal inheritance, and historical grafting practices specifically bridging Calabrian and Basilicatan olive populations remains scarce. In this study, we aimed to characterize the genetic diversity of olive accessions from Calabria and Basilicata using a robust set of SSR markers (Carriero et al., 2002; Marra et al., 2013). Furthermore, by systematically evaluating both canopy and rootstock genotypes, we sought to investigate different maternal lineages and distinct evidence of historical grafting practices among the analyzed genotypes (Mousavi et al., 2024; Valeri et al., 2022). We studied all these samples by developed markers to assign each canopy genotype to its compatibility group (Mariotti et al., 2020). The findings presented herein aim to provide novel insights into the historical management, reproductive structure, and genetic makeup of olive in southern Italy, offering valuable resources for future breeding programs and the conservation of Mediterranean agrobiodiversity.

## 2 Materials and methods

### 2.1 Plant material and DNA extraction

**Figure 1:**
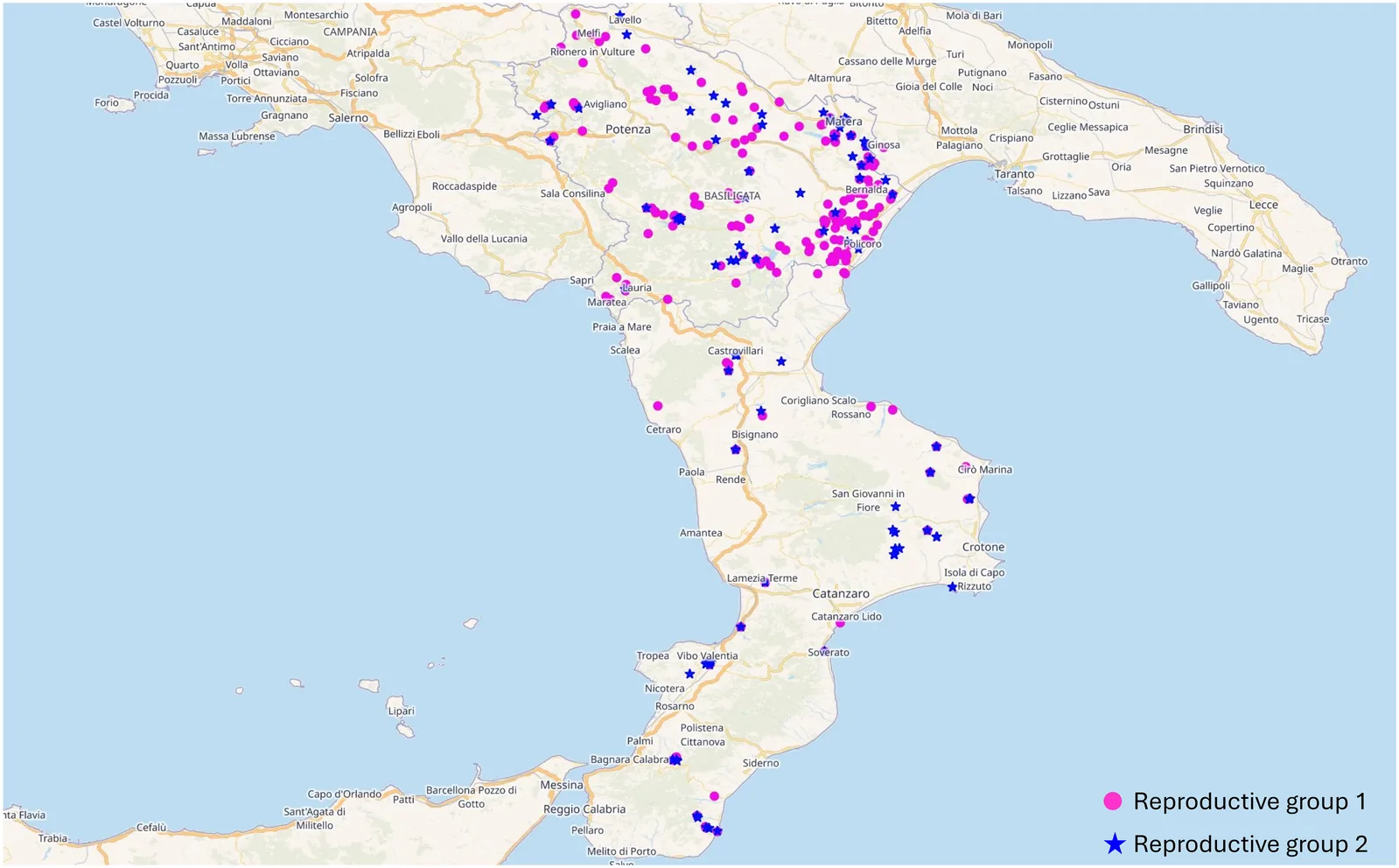
Geographical distribution of the 347 olive trees sampled from the Basilicata and Calabria regions. Circles represent individuals in reproductive group 1, while stars denote those in reproductive group 2.

A total of 347 olive trees were sampled from the Basilicata and Calabria regions of Italy (**Figure 1**). This selection comprised major and minor cultivars, unknown genotypes, and 172 monumental trees which were defined by a trunk diameter exceeding 1.0 m at a height of 130 cm from the soil. Given that many olive trees in Mediterranean regions, particularly ancient specimens, have been grafted onto different varieties or wild olives (Mousavi et al., 2024; Valeri et al., 2022), we screened for potential grafting. To achieve this, leaf samples were collected separately from the canopy and the rootstock of 117 specific trees, resulting in a total of 464 samples (**Supplementary Table 1**).

Genomic DNA was extracted from leaf tissue using the Zymo Research Mini Plant/Seed DNA Extraction Kit, following the manufacturer’s instructions. Tissue maceration was performed using a HOMEX XS homogenizer (Bioreba AG, Reinach, Switzerland) with its original extraction bags. Finally, DNA quantity and quality were assessed using a NanoPhotometer^®^ NP80 (IMPLEN, DE).

### 2.2 Nuclear and chloroplast marker applied to olive genotypes

To determine and verify the identity of the accessions, all samples were genotyped using standard dinucleotide SSR markers, which are widely utilized for cultivar characterization in olive germplasm collections (El Bakkali et al., 2019; Trujillo et al., 2014). Ten highly polymorphic markers, previously identified as the best-performing loci, were selected: DCA3, DCA5, DCA9, DCA16, DCA18, EMO-90, GAPU71B, GAPU101, GAPU103A, and UDO99-043. Forward primers were labeled at the 5’ end with VIC, PET, or NED fluorophores. The chromosomal positions for each SSR marker are provided in Valeri et al. (2022).

To determine the maternal inheritance of the samples, chloroplast genotyping was performed using cp-SSR markers p10–13, which encompass SSR, SNP, and indel variations (Mariotti et al., 2010; Besnard et al., 2011). PCR amplifications were conducted according to the method described by Mousavi et al. (2017). To visualize the alleles’ peaks, fluorescently labeled fragments were resolved via capillary electrophoresis on a SeqStudio Genetic Analyzer (Thermo Fisher Scientific, USA) using the GeneScan™ 500 LIZ internal size standard (Thermo Fisher Scientific, USA). Data analysis was performed using GeneMapper™ Software v.6 (Thermo Fisher Scientific, USA). Allele binning was performed by systematically rounding the raw, fractional fragment lengths obtained from capillary electrophoresis (e.g., 124.3 bp) into discrete integer sizes (e.g., 124 bp). This standardization ensures that genetic profiles can be accurately grouped and compiled into the final SSR allele matrix (**Supplementary Table 2**). Subsequently, strict cultivar matching criteria were applied: genotypes were classified as identical and assigned to a known reference cultivar or a specific clonal group, only if they exhibited a perfect allelic match across all 10 evaluated SSR loci.

### 2.3 Reproductive group assignment

Based on the recent findings of Castric et al. (2024), a specific primer pair was developed to target the *GA2ox-S* gene. The sequences for the primers are Forward: 5’-ATAGTCGAGGCCTGCAAAGA-3’ and Reverse: 5’-ACGACTTGAGCATCCTGTGA-3’. As established in the referenced study, this marker specifically identifies a gene fragment associated with reproductive group G1; consequently, successful amplification yielding an expected amplicon size of 314 bp indicates the presence of the G1 determinant, whereas its absence suggests group G2. The marker was rigorously validated by comparing the genetic results with phenotypic data obtained from previously published stigma tests. This validation dataset included dozens of well-characterized cultivars alongside 112 progenies derived from a controlled cross between ‘Leccino’ (G1) and ‘Dolce Agogia’ (G2) (Mariotti et al., 2020). The molecular assignment provided a clear, binary separation of the two reproductive groups that perfectly corroborated the known phenotypes. To monitor the reliability of the PCR reactions, strict internal controls were used during every amplification run. A negative control containing no template DNA was included to monitor for non-specific products and contamination. A positive control using DNA from cultivar ‘Leccino’ (a known G1 genotype) and a negative biological control using a known G2 cultivar (‘Dolce Agogia’) were also included in every run to verify the success of the PCR reaction. To rigorously manage failed amplifications and assure internal DNA quality, the 100% successful amplification of all 10 nuclear SSR loci for every individual sample (as detailed in Section 2.2) served as an absolute internal quality control. This confirmed that the absence of *GA2ox*-S amplification was a true G2 genetic signal rather than a consequence of poor DNA template quality or PCR failure. PCR amplifications were performed in a 12.5 µL reaction volume containing 25 ng of genomic DNA, 10× PCR buffer, 200 μM of each dNTP, 10 pmol of each forward and reverse primer, and 1 U of Q5 High-Fidelity DNA Polymerase (New England Biolabs, Ipswich, MA, USA).

The thermal cycling conditions consisted of an initial denaturation at 95 °C for 5 min, followed by 25 cycles of 95 °C for 25 s, 60 °C for 30 s, and 72 °C for 25 s, with a final elongation at 72 °C for 10 min. Amplicons were initially screened by electrophoresis on 2% agarose gels. Subsequently, fluorescently labeled fragments were resolved and precisely sized via capillary electrophoresis on a SeqStudio Genetic Analyzer using the GeneScan™ 500 LIZ internal size standard. Data were processed and analyzed using GeneMapper™ Software 6 (Thermo Fisher Scientific, USA).

### 2.4 Frequency analysis, evolutionary tree and Bayesian analysis

Genotypes with unique profiles, excluding genotypes identical to the known cultivars, were selected for frequency analysis. The following population-level genetic statistics were calculated using GenAlEx 6.5 (Peakall and Smouse, 2012): number of alleles (*Na*), number of effective alleles (*Ne*), observed heterozygosity (*Ho*), expected heterozygosity (*He*), and fixation indices (*F*). To determine the genetic uniqueness of each accession and quantify redundancy, the polymorphic information content (PIC) and the frequency of potential null alleles (F_null_) were calculated for each microsatellite locus using CERVUS v.3.0.7 software (Marshall et al., 1998). Furthermore, GenAlEx 6.5 was employed to estimate genetic similarity using Nei’s unbiased algorithm to differentiate the studied regional genotypes from worldwide cultivars. To further evaluate population differentiation and visually cluster the genetic relationships among the Southern Italian and worldwide populations, an Analysis of Molecular Variance (AMOVA) and a Principal Coordinate Analysis (PCoA) were also performed utilizing GenAlEx 6.5.

The SSR data from the current study were compared with a representative sample of 384 cultivars from across the Mediterranean and beyond (El Bakkali et al., 2019; Mousavi et al., 2017; Trujillo et al., 2014) here representing the ‘World’ population, resulting in a total dataset of 849 genotypes (**Supplementary Tables 1**, **3**). To infer evolutionary relationships, a phylogenetic tree was constructed in MEGA7 (Kumar et al., 2016) using the neighbor-joining (NJ) method, with branch lengths expressed in the same units as evolutionary distances.

Genetic structure was further analyzed using STRUCTURE 2.3.4 software (Pritchard et al., 2000). To avoid statistical bias caused by the overrepresentation of widespread clonal groups, this analysis utilized strictly the unique genotypes identified in this study alongside the 384 worldwide reference cultivars. The analysis utilized an admixture model with independent allele frequencies, running 100 iterations for each *K* value (ranging from *K* = 1 to 10). Each run consisted of a 100,000-step burn-in period followed by 50,000 Markov Chain Monte Carlo (MCMC) repetitions. The most likely value of K was subsequently estimated using StructureSelector (Li and Liu, 2018). To ensure the robustness of the clustering, comprehensive STRUCTURE diagnostics, specifically ΔK values, mean LnP(K), and variance among runs, were systematically evaluated and are provided. Furthermore, replicate convergence across the 100 iterations was verified using the CLUMPAK algorithm integrated within StructureSelector, ensuring stable and aligned final cluster assignments (**Supplementary Table 4**).

Finally, a Chi-square test of independence was performed to statistically evaluate the significance of the observed differences in the frequencies of the G1 and G2 reproductive groups between the Calabrian and Basilicatan populations.

## 3 Results

### 3.1 Genetic diversity characterized according to SSR and cp-SSR markers

**Figure 2:**
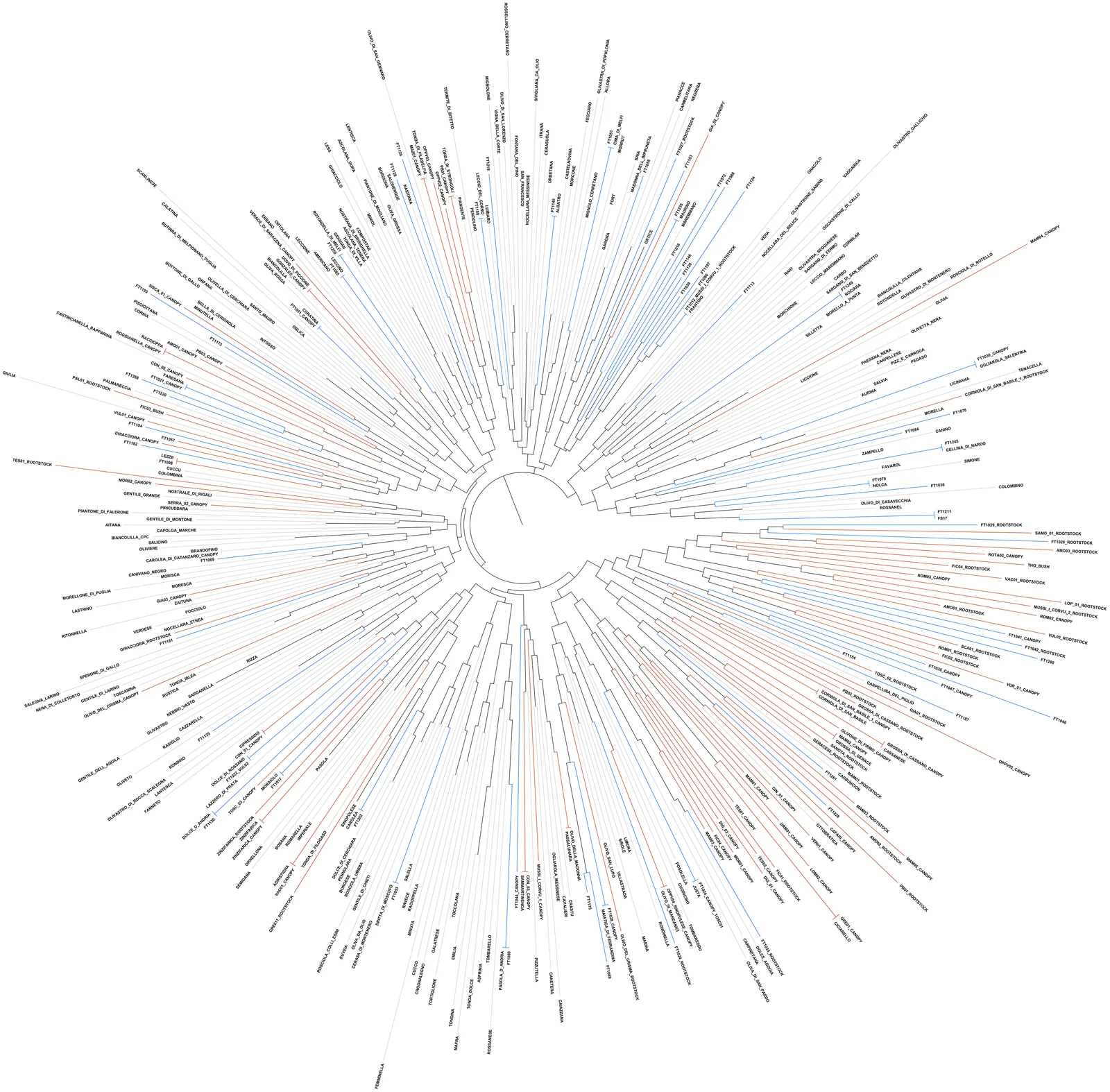
Phylogenetic relationship of the studied genotypes compared to 238 Italian cultivars. Branches are color-coded by region: red for Calabria and blue for Basilicata.

A total of 464 physical samples were successfully genotyped in this study. A comprehensive breakdown of the sampling and identification flow is provided in **Table 1**. Among these, 293 samples (63.1%) displayed genetic profiles identical to known reference cultivars: 38 Italian, one Greek (‘Kalamata’), and one French (‘Picholine de Languedoc’). A significant portion of the collection (171 samples) consisted of distinct genetic profiles from those included in the large database used for this work, with a higher prevalence in Calabria (123) compared to Basilicata (47). Notably, although it was expected to find many uncatalogued genotypes in the rootstocks, numerous were found in the canopies as well, suggesting an important reservoir of traditional germplasm in these regions (**Supplementary Table 1**). In 34 instances, the canopy genotype differed from the corresponding rootstock, consistent with historical grafting practices. Within these 171 uncatalogued samples, 17 clonal groups were identified, ultimately representing 114 distinct uncatalogued genotypes. Interestingly, certain genotypes previously thought to be unique were found to be identical despite significant geographical separation; for instance, ‘FT1103’ from Basilicata shared an identical genetic profile with ‘MUSSI_I_CORVU_2’ from Calabria, nonetheless a straight-line distance of more than 280 km between the two trees. Similar instances of clonal matches were observed within the individual regions as well. In Basilicata, the genetic profile of ‘FT1069’ was found to be identical to ‘FT1225’ and ‘FT1230’. Likewise, among the Calabrian genotypes, ‘FIC 01’ was identical to ‘FIC 02’, ‘TES 01’, and ‘SCA 01’ (**Supplementary Figure 1**). As illustrated in the evolutionary dendrogram, specific sub-clusters are predominantly or exclusively composed of rootstock genotypes from both regions, clearly demarcating them from canopy clades (e.g., the shared branch grouping rootstocks from ‘FT1042’, ‘AMO03’, ‘VUL03’, ‘FIC 04’, and ‘LOP 01’). Neighbor-Joining (NJ) analysis (**Figure 2**; **Supplementary Figure 2**) revealed that the genotypes are distributed across the entire phylogram, suggesting a broad genetic base rather than narrow lineages. Furthermore, the samples did not cluster by geographic origin; Calabrian and especially Basilicatan genotypes were heavily interspersed. Many canopy samples from both regions grouped with standard Italian reference cultivars, such as ‘Frantoio’ (known in Basilicata region as Ogliarola del Bradano), ‘Moraiolo’, or ‘Carolea’.

**Table 1:** Summary of sample collection, genotyping success, and genetic identification.

Parameter	Count	Description/notes
Sampled trees	347	Total number of individual olive trees sampled across Calabria and Basilicata.
Canopy samples	347	Leaf samples were collected from the top-graft/canopy of each tree
Rootstock samples	117	Leaf samples were successfully collected from the basal shoots/rootstocks of specific trees
Total collected samples	464	Total combined physical samples (347 canopies + 117 rootstocks).
Successfully genotyped	464	Total number of samples that successfully yielded a full SSR profile.
Missing/Failed samples	0	Samples that failed to amplify or were discarded
Cultivar matches	293	Number of successfully genotyped samples identical to known reference cultivars
Unique genotypes	171	Number of successfully genotyped samples that did not match known reference cultivarscatalogue
Distinct uncatalogued genotypes	114	The true number of unique genetic profiles found among the 171 uncatalogued samples (after grouping identical clones)
Confirmed grafting events	34	Instances where the canopy genotype differed from its corresponding rootstock genotype.

When compared the same set against the ‘World’ population (described in the material and methods section), the Southern Italian genotypes remained integrated within the broader Mediterranean pool. Calabrian and Basilicatan canopies frequently shared branches with international varieties like ‘Koroneiki’, ‘Gordal Sevillana’, ‘Empeltre’, and ‘Lucques’. However, these regional samples were notably absent from the Western Mediterranean cluster, which contains major commercial varieties from Spain (e.g., ‘Manzanilla de Sevilla’, ‘Picual’, and ‘Hojiblanca’). A similar absence was noted in the Middle Eastern cluster, representing Eastern Mediterranean and Iranian germplasm, with only rare exceptions such as ‘Fic 03’ or ‘Gia 02 Canopy’ grouping near its periphery (**Supplementary Figure 3**).

Genetic diversity indices (**Table 2**) revealed exceptionally high Polymorphism Information Content (PIC), with most loci exceeding 0.80. Loci DCA9, DCA16, and UDO-043 emerged as the most informative markers. Specifically, DCA16 in the Calabria population reached the highest PIC value (0.94). No significant deviations from Hardy-Weinberg equilibrium were observed in Basilicatan and Calabrian samples while four out of ten SSRs (DCA18, EMO90, GAPU71 and GAPU103) significantly deviated from HW equilibrium in the “World” population. *F*_null_ values were negligible, confirming the absence of null allele artifacts and the authenticity of the observed high heterozygosity.

**Table 2:** Indices of genetic diversity of 464 samples for each SSR locus: number of alleles (Na), number of effective alleles (Ne), Shannon’s information index (I), observed heterozygosity (Ho), expected heterozygosity (He), fixation index (F), Polymorphism Information Content (PIC), deviation from Hardy Weinberg equilibrium and Frequency of null alleles were reported for each analysed locus.

Population	Locus	Na	Ne	I	Ho	He	F	PIC	HW	F (null)
Basilicata	DCA3	11.00	6.85	2.08	0.85	0.85	-0.001	0.83	ND	0.00
	DCA5	11.00	2.94	1.55	0.65	0.65	0.01	0.63	NS	0.02
	DCA9	24.00	13.24	2.83	0.89	0.92	0.04	0.92	ND	0.02
	DCA16	25.00	11.31	2.77	0.94	0.91	-0.03	0.90	ND	-0.01
	DCA18	14.00	6.06	2.04	0.94	0.83	-0.12	0.81	NS	-0.06
	GAPU71B	7.00	4.74	1.67	0.85	0.78	-0.08	0.65	NS	0.05
	EMO90	6.00	3.23	1.40	0.62	0.69	0.09	0.75	NS	-0.04
	GAPU101	10.00	6.28	1.98	0.84	0.84	0.00	0.82	ND	0.00
	GAPU103A	20.00	9.41	2.49	0.72	0.89	0.18	0.88	ND	0.10
	UDO-043	20.00	12.21	2.69	0.81	0.91	0.11	0.91	ND	0.06
Calabria	DCA3	15.00	11.34	2.55	0.86	0.91	0.06	0.90	ND	0.03
	DCA5	11.00	4.95	1.95	0.81	0.79	-0.01	0.78	NS	0.00
	DCA9	23.00	15.58	2.92	0.97	0.93	-0.03	0.93	ND	-0.01
	DCA16	33.00	18.09	3.13	0.97	0.94	-0.02	0.94	ND	-0.01
	DCA18	15.00	5.58	2.05	0.77	0.82	0.05	0.80	NS	0.02
	GAPU71B	6.00	2.88	1.35	0.55	0.65	0.16	0.61	NS	0.08
	EMO90	7.00	4.19	1.59	0.79	0.76	-0.03	0.72	NS	-0.01
	GAPU101	14.00	7.76	2.30	0.88	0.87	-0.01	0.85	ND	0.00
	GAPU103A	15.00	7.43	2.19	0.74	0.86	0.14	0.85	ND	0.07
	UDO-043	20.00	8.19	2.48	0.83	0.87	0.05	0.86	NS	0.02
World	DCA3	15.00	8.55	2.33	0.89	0.88	-0.01	0.87	NS	0.00
	DCA5	15.00	2.86	1.60	0.63	0.65	0.02	0.63	NS	0.00
	DCA9	27.00	12.15	2.75	0.89	0.91	0.02	0.91	NS	0.01
	DCA16	32.00	9.72	2.60	0.85	0.89	0.04	0.88	NS	0.02
	DCA18	18.00	6.64	2.13	0.89	0.84	-0.04	0.83	*	-0.02
	GAPU71B	13.00	3.67	1.63	0.69	0.72	0.04	0.69	**	0.01
	EMO90	18.00	5.32	1.88	0.85	0.81	-0.05	0.78	*	-0.03
	GAPU101	17.00	7.83	2.24	0.91	0.87	-0.04	0.85	NS	-0.02
	GAPU103A	29.00	9.00	2.53	0.75	0.88	0.14	0.87	**	0.07
	UDO-043	28.00	10.40	2.63	0.84	0.90	0.06	0.89	NS	0.03
Total	Mean	17.30	7.95	2.21	0.81	0.83	0.02			
	SE	1.387	0.70	0.09	0.01	0.01	0.01			

NS, not significant deviation from Hardy Weinberg equilibrium; *significant deviation from HW after Bonferroni correction (p<0.05), **(p<0.01), ***(p<0.001).

In Basilicata, the number of alleles (*N_a_*) ranged from 6 (EMO90) to 25 (DCA16). Observed heterozygosity (*H_o_*) peaked at 0.94 (DCA16 and DCA18). Fixation indices (*F*) indicated an excess of heterozygotes at several markers, particularly DCA18 (-0.13) and GAPU71B (-0.08). Calabria exhibited even higher variation; DCA16 was remarkably polymorphic with 33 alleles, surpassing the diversity found in the “World” reference population (32 alleles). Calabria also demonstrated a higher number of effective alleles (*N_e_*) for DCA9 (15.58) and DCA16 (18.09) compared to global averages. Mean *F* values across all groups remained near zero, suggesting a balanced population structure and low inbreeding.

### 3.2 Some Calabrian local olives are putative polyploids

Among all analyzed samples, six (originating from the canopy and rootstock of three specific olive trees) growing in Calabria exhibited more than two alleles for eight out of the ten amplified SSRs. This was an unexpected finding for a species typically considered diploid, to be putatively polyploidy. To validate these results, DNA was extracted from these samples three separate times; additionally, both the amplification and sequencer runs were performed in duplicate. Three distinct genetic profiles were observed, and all analyzed canopies were found to be genetically identical to their respective rootstocks (**Supplementary Table 5**; **Supplementary Figure 4**).

### 3.3 Chloroplast lineages and maternal inheritance

**Figure 3:**
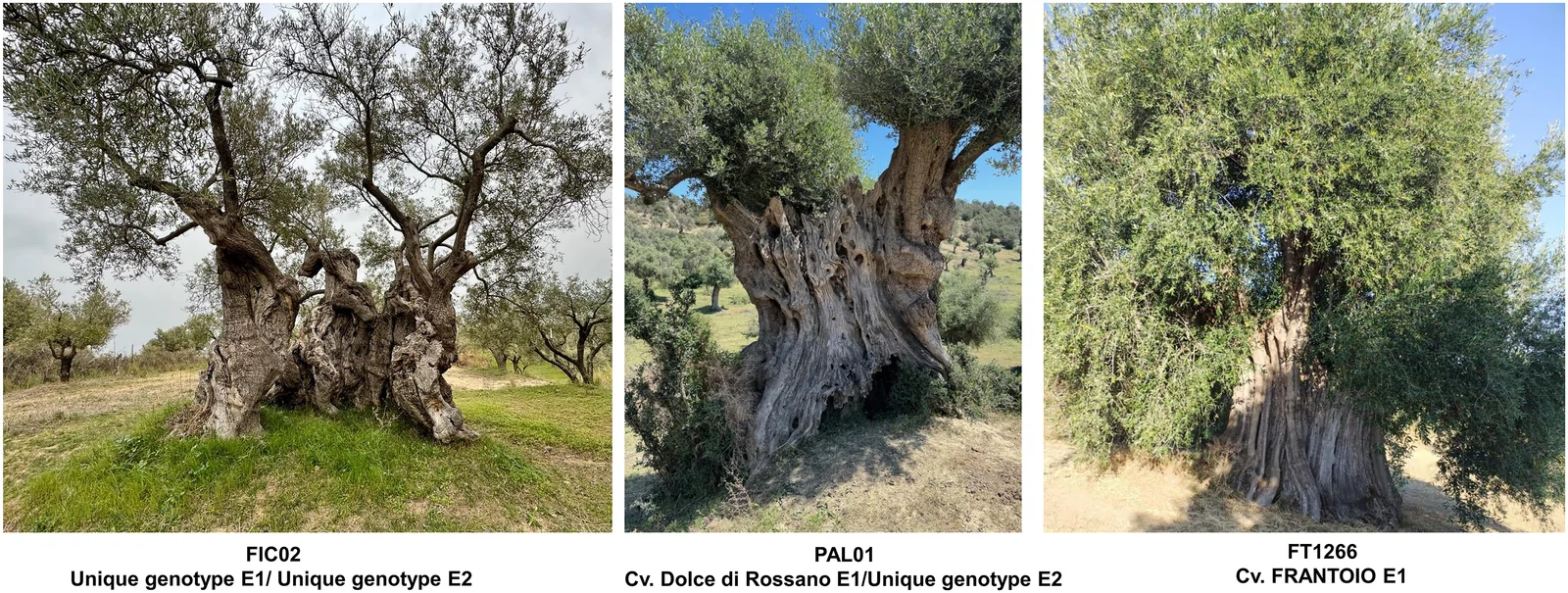
Genetic lineages of monumental Calabrian and Basilicatan olive trees. This analysis details the canopy/rootstock lineages of grafted genotypes versus the uniform lineages of non-grafted trees. The E1 lineage is most common in cultivated varieties (var. *europaea*), while the E2 lineage remains the standard for wild types (var. *sylvestris*).

Analysis of cpSSR markers identified two distinct lineages: E1 and E2. The E2 lineage was found in 48 samples (including 21 rootstocks). Canopy genotypes identical to ‘Lezze’ (known in Basilicata area as “Tarantina”) and ‘Picholine de Languedoc’, as well as several unique genotypes (e.g., FT1041, GIA 02, ROM02), were assigned to the E2 lineage. A total of 13 monumental trees within the sampled population were identified as having a trunk diameter exceeding 200 cm. Almost all of them, except FT1266 Frantoio cultivar, were from Calabria region. Correlation of these monumental trees with their maternal lineages reveals a strong preservation of the Central-Western Mediterranean E2 lineage at their rootstock, six out of 13 trees. The Eastern Mediterranean E1 lineage was present in the rootstocks of seven monumental trees (**Figure 3**; **Supplementary Figure 5**).

### 3.4 Genetic differentiation of studied genotypes

**Figure 4:**
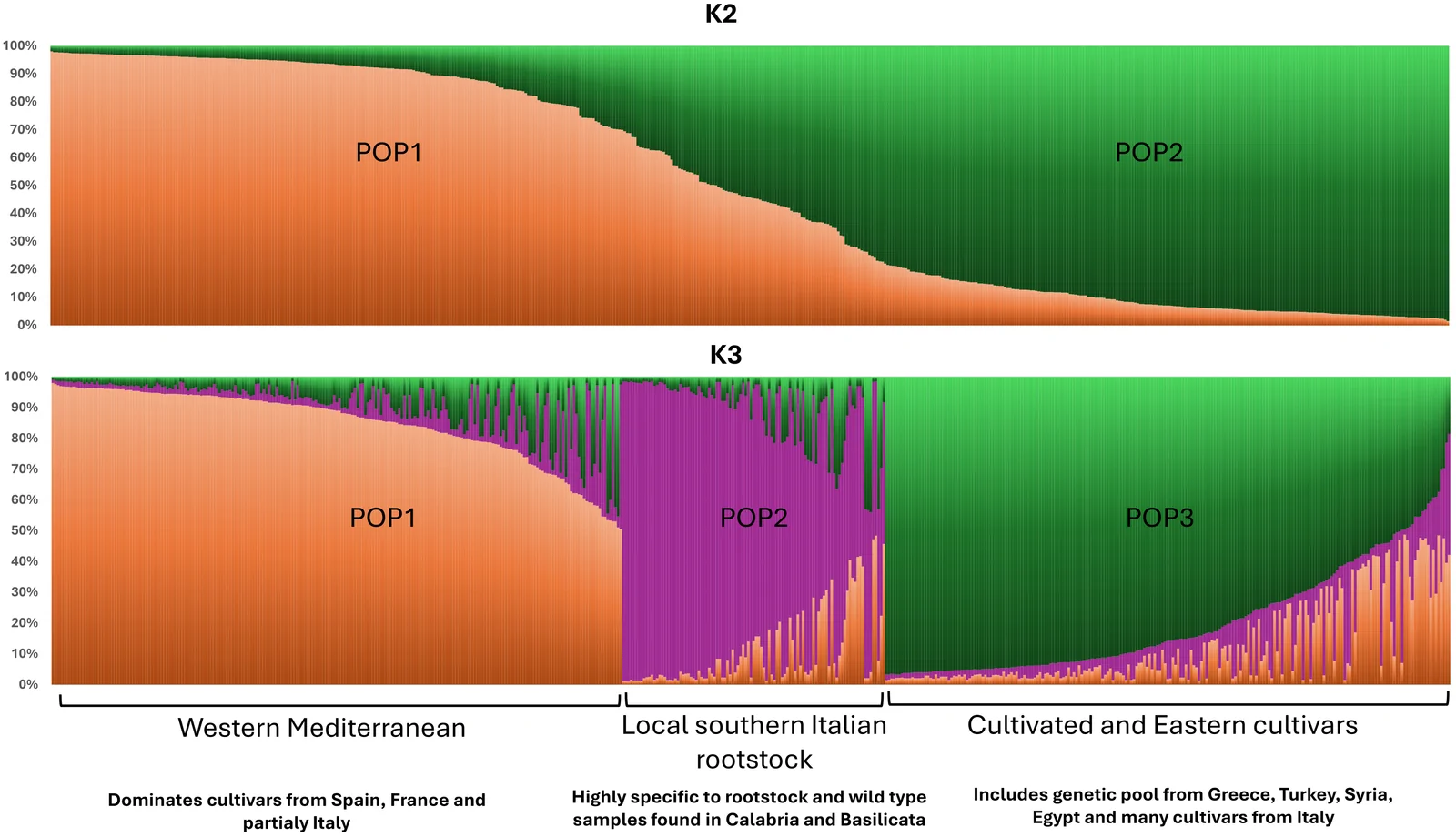
Genetic structure of the unique genotypes analyzed in this study alongside 384 worldwide cultivars, based on SSR markers. Colors represent the three detected populations, while samples displaying multiple colors indicate an admixed genetic composition.

Nei’s unbiased genetic similarity (**Table 3**) showed that both regions are most closely related to the broader Italian pool (0.91 for Basilicata; 0.86 for Calabria), with a strong reciprocal similarity (0.81). Beyond Italy, Basilicata showed affinity with Greece and Algeria, while Calabria aligned with Malta and again Algeria. Both regions were most genetically distant from Eastern populations in Turkey and Iran. To statistically quantify the partitioning of genetic variation, an Analysis of Molecular Variance (AMOVA) was conducted. The AMOVA revealed that most of the genetic variation (96%) was partitioned within individuals, whereas only 3% of the variance was attributed to differences among populations (Fst = 0.029, p = 0.001). The inbreeding coefficient (Fis = 0.013) and overall fixation index (Fit = 0.042) were remarkably low, while the estimated gene flow was high (Nm = 8.357). Together, these statistics confirm a highly outcrossing mating system with extensive historical gene flow and extremely low genetic differentiation among the evaluated Mediterranean populations (**Supplementary Table 6**). To visualize these genetic relationships without relying on predefined population models, a Principal Coordinate Analysis (PCoA) was performed. The first two principal coordinates explained 5.98% and 4.56% of the total variance, respectively. The resulting scatter plot visually corroborated the genetic affinities observed in the Nei’s similarity matrix, demonstrating that the Basilicata and Calabria genotypes do not form an isolated genetic island, but rather cluster in close spatial proximity to specific international pools, particularly aligning with accessions from Algeria and Greece (**Supplementary Figure 6**). A population structure analysis of data on the all-unique genotypes together with 385 world genotypes was evaluated using the Evanno method. The ΔK diagnostic identified K = 3 as the optimal number of populations (highest peak ΔK = 7.53), with a secondary sub-structural signal at K = 2 (ΔK = 4.40, **Supplementary Figure 7**). At K = 2, the analysis assigned individuals to a population for values above 50%, except in cases of intermixed genotypes (**Figure 4**). POP1a, which is mostly assigned to local and central Mediterranean dominates the Calabrian and Basilicatan samples. Both local wild rootstocks (such as ‘TOSC_02’ and ‘FT1026’) and local cultivated canopies (such as ‘FT1047’) show extremely high genetic assignments, often over 97%, to this group. It also encompasses widespread standard Italian cultivars like ‘Frantoio’, ‘Ogliarola Salentina’, and ‘Ottobratica’. POP2a which assigned to Western Mediterranean and international cultivars, particularly from Spain, France, and North Africa. Widespread varieties such as ‘Gordal Sevillana’ (98.5% POP2a), ‘Picual’ (97.3%), ‘Manzanilla de Sevilla’ (96.5%), and ‘Lucques’ belong almost entirely to this group, showing very little admixture with the local Southern Italian pool.

**Table 3:** Nei unbiased genetic similarity between Basilicata and Calabria province versus the most producer olive Countries.

Population	Basilicata	Population	Calabria
Italy	0.91	Italy	0.86
Greece	0.85	Malta	0.84
Algeria	0.82	Algeria	0.84
Calabria	0.81	Basilicata	0.81
France	0.77	Greece	0.80
Malta	0.76	Syria	0.77
Spain	0.76	Spain	0.77
Tunisia	0.71	France	0.73
Syria	0.70	Tunisia	0.71
Egypt	0.69	Egypt	0.68
Türkiye	0.67	Türkiye	0.66
Iran	0.59	Iran	0.63

Fully supported by the primary ΔK peak, the structure analysis for three populations (K = 3) demonstrates that the large Italian/local pool from the K = 2 analysis splits, revealing a much more distinct and biologically informative structure. POP1b cluster remains consistent with POP2a from the K = 2 analysis. It is dominated by major commercial Spanish and international cultivars, such as ‘Gordal Sevillana’ (98% POP1b) and ‘Picual’ (96.3%). POP2b was an additional cluster that groups the native, wild genetic pool of the region. It is overwhelmingly populated by the uncultivated rootstocks of Basilicata and Calabria (e.g., ‘FT1026’, ‘LOP_01’, ‘VUL03’, and ‘MUSSI_I_CORVU_2’, all of which show >96% assignment to this specific genetic cluster). This suggests that the monumental rootstocks represent a localized lineage genetically distinct from standard commercial varieties. POP3b encompasses the established Italian and Mediterranean reference canopies (e.g., ‘Frantoio’ at 96% POP3b, ‘Maiatica di Ferrandina’, and ‘Moraiolo’). The cultivated canopies genotypes from Calabria and Basilicata frequently fall into this group, providing genetic evidence consistent with the historical agricultural practice of farmers grafting these widespread, successful commercial canopies (POP3b) onto wild rootstocks (POP2b) (**Figure 4**). The K = 3 population structure analysis reveals highly admixed local genotypes in Calabria and Basilicata, indicating potential hybridization patterns across the Western Mediterranean (POP1b), Local Rootstock (POP2b), and Cultivated Central/Eastern Mediterranean (POP3b) pools.

First, direct introgression between the Western Mediterranean and local rootstocks is evident in regional canopies like the Calabrian ‘Verace di Saracena’ and Basilicatan ‘FT1008’ and ‘FT1182’. Second, admixture bridging the indigenous wild rootstocks and established cultivated Mediterranean pools appears in Basilicatan canopies such as ‘FT1245’ and ‘FT1219’. Third, complex three-way gene flow spanning all three ancestral pools is observed in specific profiles like the Calabrian ‘Ghiacciora Rootstock’ and the Basilicatan canopy ‘FT1129’. Overall, these intermixed profiles provide evidence of continuous, multi-generational outcrossing between autochthonous wild lineages and diverse introduced elite clones within the Southern Italian landscape.

### 3.5 Reproductive group assignment

Amplification of extracted DNA from each canopy through the dominant marker built within the *GA2ox* gene provided, as expected, the presence of the two reproductive groups in both Italian regions. 221 samples belonged to the G1 reproductive group, and 126 samples belonged to the G2 reproductive group. A Chi-square test of independence revealed a highly significant difference in the frequencies of reproductive groups between the two regions (χ² = 42.90, p < 0.001). In the Basilicata region the G1 reproductive group is dominant (185 G1 vs. 64 G2). This is driven by the high prevalence of widely propagated commercial and historical cultivars that belong to G1, such as ‘Frantoio’, ‘Faresana’ (known in Basilicata region as Acerenza), ‘Justa’, ‘Raccioppa’, and ‘Maiatica di Ferrandina’. While G2 is present (found in cultivars like ‘Ogliarola Salentina’, ‘Coratina’, and ‘Lezze’), it makes up a much smaller portion of the Basilicatan canopies. In contrast, the G2 reproductive group is highly prevalent and more common among the Calabrian samples (62 G2 vs. 36 G1). Many of the local and unique Calabrian canopies are classified as G2, including the entire ‘FIC’ series, almost the entirety of the ‘GRE’ series (identified as ‘Ciciarello’), the ‘SCA’ series, the ‘MAM’ series (excluding MAM05), and various unique genotypes like ‘AMO01’, ‘PB01’, and ‘ROM01’. G1 is still present in Calabria (e.g., ‘VUL’ series, ‘LOM’ series, and ‘OPPV04’), but G2 makes up the larger share (**Figure 1**; **Supplementary Table 1**).

## 4 Discussion

### 4.1 Monumental trees as vital reservoirs of uncatalogued traditional germplasm

The detection of 114 genetically distinct profiles in our study, 72 in Calabria and 42 in Basilicata, identifies Southern Italy as a vital center for olive genetic resources. This extraordinary rate of uncatalogued diversity aligns with recent molecular surveys of monumental trees across the Mediterranean, which consistently demonstrate that historic groves act as *in situ* reservoirs for uncharacterized genetic diversity. For instance, in a genetic analysis of centennial olive trees in southern Spain, only 9.6% of ancient genotypes matched currently catalogued cultivars, indicating that the vast majority represent unknown traditional genotypes that have evaded modern characterization (Díez et al., 2011). Similarly, recent investigations of monumental olive trees on Capri Island, some with an estimated age of 932 years after radiocarbon dating, revealed unique genetic profiles that did not match any of hundreds of worldwide reference varieties (Mousavi et al., 2024). Even singular patriarchal trees, such as the >700-year-old “Olivo della Strega” in Tuscany, have been shown to harbor completely unknown genotypes alongside known varieties like ‘Frantoio’ due to historic grafting (Petruccelli et al., 2021). Our discovery that the oldest surviving monumental trees, (such as FT1266 ‘Frantoio’ with trunk diameter of 240 cm and GRE03 ‘Ciciarello’ with trunk diameter more than 300 cm) preserve such high observed heterozygosity (*H_o_* = 0.82) and allelic richness supports the concept that long-term vegetative propagation successfully maintains elite, highly heterozygous crosses. However, as modern orchards increasingly rely on a restricted number of commercial varieties, this unique, locally adapted germplasm faces severe risk of genetic erosion, mirroring trends observed in ancient versus centennial Portuguese ‘Galega vulgar’ populations where rare allelic combinations have been lost over time (Sales et al., 2021). The individuation of identical clonal groups across significant geographical distances provides compelling evidence of widespread human-mediated dispersal and ancient agricultural exchange (Mousavi et al., 2017; Mariotti et al., 2021). Because the olive tree’s diallelic self-incompatibility system enforces strict outcrossing that naturally generates highly recombinant, novel genotypes through sexual reproduction, the presence of exact genetic matches separated by vast distances strongly refutes natural seed dispersal and instead confirms deliberate vegetative propagation by early agrarian societies (Mariotti et al., 2021). Early farmers systematically selected and cloned these specific, highly heterozygous genotypes to reliably reproduce their superior phenotypic traits, such as enhanced resilience to environmental stressors or exceptional agronomic performance (Díez et al., 2011; Barazani et al., 2014). The active, cross-regional dissemination and long-term *in situ* maintenance of these identical, uncatalogued clones underline their profound historical importance, demonstrating that they were highly prized agricultural assets cultivated, shared, and preserved by early communities long before the formal cataloging of modern commercial varieties (Díez et al., 2011; Mousavi et al., 2024).

Among the unique genotypes identified in the Calabria region, the discovery of six samples, derived from the canopy and rootstock of three specific individuals (‘GRE05’, ‘RAN01’, and ‘RAN02’), offers genetic evidence suggesting putative polyploidy in indigenous Italian olives. These specimens exhibited more than two alleles at eight out of ten amplified SSR loci, a finding that challenges the standard view of the *Olea europaea* complex. Generally, this species is regarded as strictly diploid; established polyploidy has traditionally been documented only in geographically sequestered taxa, such as the hexaploid subsp. *maroccana* from the Agadir Mountains and the tetraploid subsp. *cerasiformis* indigenous to Madeira (Besnard et al., 2012; Julca et al., 2023). While spontaneous triploidy has been noted as a rare phenomenon within the fragmented and isolated Saharan subsp. *laperrinei* (Besnard et al., 2012), its presence within the highly admixed Mediterranean germplasm is a notable observation. Research further revealed that the genetic signatures of the Calabrian canopies were identical to their corresponding rootstocks, indicating that these putative polyploid individuals are likely ungrafted and self-rooted. Their genetic consistency implies that, much like the adaptive strategies seen in Saharan triploids (Besnard et al., 2012), these distinctive Calabrian putative polyploids have endured over time primarily through their highly heterozygous, multi-allelic profiles within the local environment.

### 4.2 Historical grafting practices: exploiting wild rootstocks for local adaptation

The population genetic analyses provide compelling statistical support for the highly admixed nature of Southern Italian olive germplasm. The exceptionally high estimated gene flow and the PCoA clustering patterns demonstrate that the Calabrian and Basilicatan populations have not evolved as isolated genetic islands. Instead, their spatial clustering and close genetic affinity with standard international pools, particularly accessions from Greece and Algeria, provide molecular evidence for continuous, human-mediated germplasm exchange across the Mediterranean Basin. This lack of regional isolation perfectly aligns with historical and archaeological records of early agricultural expansion, particularly the dynamic trade driven by the 8th-century BC Greek colonies. Within this admixed landscape, our results provide genetic evidence consistent with historical grafting practices, which have shaped the genetic landscape of Southern Italy. The *K* = 3 population structure analysis partitioned the native, uncultivated rootstocks into a distinct and localized lineage (POP2b), while their respective cultivated canopies grouped with widespread Central and Eastern Mediterranean commercial varieties (POP3). Furthermore, the discovery that 35 of our uncatalogued unique genotypes are localized exclusively within rootstocks demonstrates that the basal portions of these trees harbor their own hidden diversity.

These findings reflect patterns observed in the Levant, providing molecular evidence that farmers systematically grafted elite clones onto diverse, locally adapted rootstocks (Barazani et al., 2014). By grafting imported or elite canopies onto resilient local wild types, a practice described as “instant domestication” (Díez et al., 2011), early farmers across the Mediterranean essentially preserved two parallel genetic pools in a single orchard. The distinct POP2b rootstocks we identified in Calabria and Basilicata are consistent with this historic agricultural strategy, safeguarding a local genetic base that evolved separately from the heavily traded commercial canopy clones.

### 4.3 Maternal lineages and the east-to-west domestication trajectory

The duality between rootstock and canopy is further reinforced by our chloroplast lineage analysis, which revealed a massive concentration of the E2 lineage in local wild rootstocks (21 samples), while the E1 lineage overwhelmingly dominates the cultivated canopies (336 samples). Remarkably, all the trees having a trunk diameter exceeding 200 cm, are considered putatively ancient monumental trees (Bernabei, 2015). This distribution provides strong molecular support for the primary East-to-West domestication model (Mariotti et al., 2023; Besnard et al., 2013).

Extensive phylogenomic and genealogical tracing suggests that primary domestication occurred in the Eastern Mediterranean, yielding highly productive, large-fruited E1 clones (Mariotti et al., 2023; Bazakos et al., 2023). As these elite eastern founders (such as the ancestors of ‘Gordal Sevillana’ and ‘Safrawi’) were disseminated westward via maritime trade, they were systematically grafted onto autochthonous Central and Western Mediterranean wild oleasters, which predominantly carry the E2 and E3 lineages (Mariotti et al., 2023; Mousavi et al., 2024). Our data are consistent with this historical sequence: the local Calabrian and Basilicatan rootstocks preserve the indigenous E2 maternal heritage, while the oldest and most successful canopies represent the imported E1 lineage. The rare emergence of the E2 lineage within a select few cultivated canopies, most notably the local ‘Lezze’ cultivar and several unique profiles, suggests to localized, secondary domestication events where indigenous Central Mediterranean wild types were occasionally selected by farmers for their unique regional traits (Tourvas et al., 2023; Mariotti et al., 2023; Valeri et al., 2022).

### 4.4 Diallelic self-incompatibility and its role in maintaining widespread heterozygosity

The AMOVA revealed that most of the genetic variation is partitioned within individuals rather than between populations, accompanied by a remarkably low inbreeding coefficient. This massive intra-individual diversity is a direct evolutionary consequence of the olive diallelic self-incompatibility (DSI) system, which enforces outcrossing and prevents localized inbreeding. Finally, the exceptional genetic diversity and excess of heterozygosity (indicated by negative mean fixation index, *F* values across markers like DCA16 and DCA18) observed in the Calabrian and Basilicatan populations are influenced by the olive tree’s unique reproductive biology, alongside historical germplasm exchange and clonal propagation. Our classification of the regional canopies into the G1 (dominant in Basilicata) and G2 (highly prevalent in Calabria) reproductive groups underscores the biological barrier that has historically prevented inbreeding.

It is now understood that olive reproduction is governed by a strict homomorphic diallelic self-incompatibility (DSI) system, which mandates outcrossing by classifying all varieties into two inter-incompatible mating groups (Mariotti et al., 2020). Recent genomic breakthroughs have demonstrated that this DSI system is controlled by a hemizygous genomic region expressing the gibberellin pathway gene *GA2ox*-S, an ancient evolutionary mechanism shared across the *Oleaceae* family (Castric et al., 2024). Because fertilization is impossible within the same group (e.g., G1 cannot pollinate G1), the system enforces obligation outcrossing between diverse genotypes in the orchard (Mariotti et al., 2021). By naturally driving up heterozygosity, and through the subsequent vegetative propagation of these successful crosses by early farmers, the DSI system contributes significantly to the generation and maintenance of the highly admixed, polymorphic genetic structure and the abundance of unique local genotypes that currently define the traditional olive groves of Southern Italy (Muzzalupo et al., 2014; Marra et al., 2013). Beyond its evolutionary role, identifying the specific reproductive group of these cultivars holds profound agronomic and breeding importance. Because successful fruit production requires cross-pollination between varieties of different SI groups, modern trends of planting mono-varietal orchards or mixing varieties with unbalanced SI groups can lead to severe pollen limitation, fruiting failure, and suboptimal yield (Mariotti et al., 2021). Therefore, determining the G1 or G2 phenotype of both established cultivars and uncatalogued putatively ancient genotypes is essential for designing effective orchard layouts that guarantee the presence of compatible pollen donors, thereby maximizing fruit set and overall production (Mariotti et al., 2020; Mariotti et al., 2021). For future breeding programs, characterizing these incompatibility groups enables early marker-assisted selection, which guides the choice of compatible parental combinations and significantly expedites the historically slow process of olive genetic improvement (Mariotti et al., 2020). Furthermore, the recent discovery that treatments targeting the gibberellic acid (GA) pathway can temporarily switch SI specificities offer novel biotechnological applications to obtain selfed progenies or secure fruit production in geographically isolated orchards where compatible pollen availability is low (Castric et al., 2024).

## 5 Conclusion

The comprehensive molecular characterization of the olive germplasm in Calabria and Basilicata reveals an exceptional reservoir of genetic diversity. By integrating nuclear SSRs, chloroplast lineage tracing, and reproductive group assignments, this study provides molecular evidence suggesting of how historical agricultural practices and unique biological mechanisms have synergistically shaped the Mediterranean olive landscape (Mariotti et al., 2023). The distinct structural partition between elite cultivated canopies (predominantly of the E1 maternal lineage) and resilient, highly localized wild rootstocks (predominantly of the E2 lineage) is consistent with the historical strategy of grafting imported or widespread commercial clones onto autochthonous wild oleasters (Barazani et al., 2014; Valeri et al., 2022; Mariotti et al., 2023).

These monumental trees serve as living witnesses to historical agrarian landscapes and act as *in situ* reservoirs for uncatalogued traditional genotypes that have otherwise been lost to modern, intensive mono-varietal agriculture (Díez et al., 2011; Mousavi et al., 2024). Because these veteran trees have already survived centuries of extreme environmental fluctuations, their genetic makeup holds potential adaptive value (Barazani et al., 2017). As modern olive growing faces escalating threats from climate change and emerging pathogens, the conservation of these unique Southern Italian genotypes is of paramount importance (Mousavi et al., 2024). Integrated conservation efforts are mandatory to protect these putatively ancient genotypes *in situ* and integrate them into *ex situ* germplasm collections to avoid irreversible genetic erosion (Díez et al., 2011; Sales et al., 2021). Ultimately, exploiting this untapped genetic diversity in future breeding programs will be important for designing resilient, locally adapted fruit tree portfolios capable of confronting future agricultural challenges.

## Data Availability

The original contributions presented in the study are included in the article/**Supplementary Material**. Further inquiries can be directed to the corresponding author.
