# Supplementary material for: Deciphering centuries of selection: genetic structure and reproductive screening in Basilicata and Calabria’s ancient olives
Source: Front Plant Sci. 2026 Aug 12;17:1887538. doi: 10.3389/fpls.2026.1887538 (PMC13506961; doi:10.3389/fpls.2026.1887538)

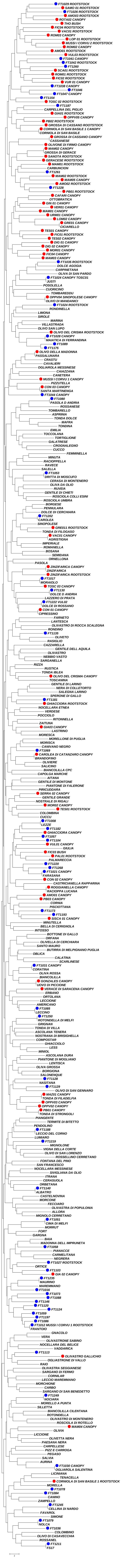

Supplement: Supplementary file 1 [file DataSheet1.pdf]
