# Supplementary material for: Deciphering centuries of selection: genetic structure and reproductive screening in Basilicata and Calabria’s ancient olives
Source: Front Plant Sci. 2026 Aug 12;17:1887538. doi: 10.3389/fpls.2026.1887538 (PMC13506961; doi:10.3389/fpls.2026.1887538)
Supplement: Supplementary file 2 [file Table5.docx]

**Table S5.** Genetic profile of the individuates polyploid samples in the Calabria region by SSR markers.

|  | **DCA3** | | **DCA5** | | | **DCA9** | | | **DCA16** | | | **DCA18** | | | **EMO90** | | | **GAPU71B** | | | **GAPU101** | | | **GAPU103A** | | | **UDO-043** | |
| --- | --- | --- | --- | --- | --- | --- | --- | --- | --- | --- | --- | --- | --- | --- | --- | --- | --- | --- | --- | --- | --- | --- | --- | --- | --- | --- | --- | --- |
| **GRE 05 C** | 235 | 243 | 194 | 202 | 206 | 203 | 203 |  | 124 | 156 |  | 171 | 173 | 175 | 188 | 194 | 198 | 124 | 126 | 130 | 185 | 193 | 207 | 150 | 156 | 174 | 170 | 174 |
| **GRE05 R** | 235 | 243 | 194 | 202 | 206 | 203 | 203 |  | 124 | 156 |  | 171 | 173 | 175 | 188 | 194 | 198 | 124 | 126 | 130 | 185 | 193 | 207 | 150 | 156 | 174 | 170 | 174 |
| **RAN01 C** | 235 | 241 | 194 | 202 | 206 | 198 | 204 | 208 | 124 | 146 | 158 | 171 | 173 | 177 | 188 | 194 | 198 | 124 | 126 | 130 | 185 | 193 | 207 | 150 | 156 | 174 | 170 | 174 |
| **RAN01 R** | 235 | 241 | 194 | 202 | 206 | 198 | 204 | 208 | 124 | 146 | 158 | 171 | 173 | 177 | 188 | 194 | 198 | 124 | 126 | 130 | 185 | 193 | 207 | 150 | 156 | 174 | 170 | 174 |
| **RAN02 C** | 235 | 241 | 194 | 202 | 206 | 190 | 198 | 208 | 124 | 146 | 158 | 171 | 173 | 177 | 188 | 194 | 198 | 124 | 126 | 130 | 185 | 193 | 207 | 150 | 156 | 174 | 170 | 174 |
| **RAN02 R** | 235 | 241 | 194 | 202 | 206 | 190 | 198 | 208 | 124 | 146 | 158 | 171 | 173 | 177 | 188 | 194 | 198 | 124 | 126 | 130 | 185 | 193 | 207 | 150 | 156 | 174 | 170 | 174 |
